# Validation of Self-Reported Information on Dental Caries in a Birth Cohort at 18 Years of Age

**DOI:** 10.1371/journal.pone.0106382

**Published:** 2014-09-09

**Authors:** Alexandre Emidio Ribeiro Silva, Ana Maria Baptista Menezes, Maria Cecília Formoso Assunção, Helen Gonçalves, Flávio Fernando Demarco, Fabiana Vargas-Ferreira, Marco Aurélio Peres

**Affiliations:** 1 Postgraduate Programme in Epidemiology, Federal University of Pelotas, Pelotas, Rio Grande do Sul, Brazil; 2 Australian Research Centre for Population Oral Health, School of Dentistry, University of Adelaide, Adelaide, Austrália; University of Washington, United States of America

## Abstract

**Objective:**

Estimate the prevalence of dental caries based on clinical examinations and self-reports and compare differences in the prevalence and effect measures between the two methods among 18-year-olds belonging to a 1993 birth cohort in the city of Pelotas, Brazil.

**Method:**

Data on self-reported caries, socio-demographic aspects and oral health behaviour were collected using a questionnaire administered to adolescents aged 18 years (n = 4041). Clinical caries was evaluated (n = 1014) by a dentist who had undergone training and calibration exercises. Prevalence rates of clinical and self-reported caries, sensitivity, specificity, positive and negative predictive values, absolute and relative bias, and inflation factors were calculated. Prevalence ratios of dental caries were estimated for each risk factor.

**Results:**

The prevalence of clinical and self-reported caries (DMFT>1) was 66.5% (95%CI: 63.6%–69.3%) and 60.3% (95%CI: 58.8%–61.8%), respectively. Self-reports underestimated the prevalence of dental caries by 9.3% in comparison to clinical evaluations. The analysis of the validity of self-reports regarding the DMFT index indicated high sensitivity (81.8%; 95%CI: 78.7%–84.7%) and specificity (78.1%; 95%CI: 73.3%–82.4%) in relation to the gold standard (clinical evaluation). Both the clinical and self-reported evaluations were associated with gender, schooling and self-rated oral health. Clinical dental caries was associated with visits to the dentist in the previous year. Self-reported dental caries was associated with daily tooth brushing frequency.

**Conclusions:**

Based on the present findings, self-reported information on dental caries using the DMFT index requires further studies prior to its use in the analysis of risk factors, but is valid for population-based health surveys with the aim of planning and monitoring oral health actions directed at adolescents.

## Introduction

Self-reported health status is considered a valid, acceptable method for the assessment of the prevalence of diseases in the general population, such as hypertension and diabetes [1], as well as risk factors, such as poor diet and lack of physical activity [2]. In the field of dentistry, self-reported information is an economically feasible option for measuring oral health conditions in population-based multidisciplinary surveys and diminishes the need for time-consuming clinical exams [3]. Moreover, self-reports have the potential to be a useful method for monitoring oral health conditions and trends over time, which is important to the planning and evaluation of public health policies. For instance, the Centers for Disease Control and Prevention in the United States of America use health information acquired from telephone surveys [4].

Health inquiries involving self-reported information indicate that characteristics related to socio-demographic aspects [5] and past disease experience [6] have a direct influence on the knowledge of individuals regarding their health, producing greater or lesser agreement between self-reports and clinically determined data. In the field of oral health, studies indicate an underestimation of periodontal disease in self-reported information. [2], [7], [8]. A systematic review on this issue demonstrates that the use of self-reported data is inadequate for information on gingivitis, but other self-reported measures of periodontal disease have proven to be valid [1], such as the occurrence of periodontal pockets. However, standardisation is required regarding the different methods used for the acquisition of self-reported information.

The literature demonstrates greater validity in self-reported information related to the use of dentures and the number of teeth in the oral cavity [2], [4], [5], [9]–[11]. However, some studies indicate a slight underestimation [2] or overestimation [2], [7] regarding the number of teeth. For measures related to tooth decay, an underestimate of the number of teeth with carious tissue has been found in self-reports [2] and reports of missing teeth should be used with the proper corrections [10]. However, with regard to the presence/absence of dental caries, studies involving adults indicate a lack of agreement with clinical measures, mainly because individuals are often unable to recognise caries [2], [6].

Few studies have been conducted on the validity of self-reported information regarding dental caries in 18-year-olds in countries of low to middle income. Therefore, the aims of the present study were to estimate the prevalence of dental caries based on clinical examinations and self-reports and compare differences in the prevalence and effect measures between the two methods among 18-year-olds belonging to a 1993 birth cohort in the city of Pelotas, Brazil.

## Materials and Methods

### Description of 1993 Pelotas birth cohort

This was the second birth cohort in the city of Pelotas (southern Brazil) and was separated from the first cohort study by 11 years. The aim was to allow the comparison of mother/child characteristics of the population and changes in the main health indicators as well as to evaluate the influence of factors related to birth and early childhood on health throughout the life cycle. Moreover, it is one of the few population-based studies to investigate oral health.

All live births recorded at hospitals in the city of Pelotas in 1993 from mothers residing in urban areas were included in this cohort. Among the 5265 women who had children in the period, 5249 (99.7%) agreed to participate in the study. Subsamples were revisited when the members of the cohort were 1, 3, and 6 months of age as well as 1, 3, 6, and 9 years of age. In 2004, when the members were 11 years of age, the entire cohort was evaluated; the same occurred in 2008 (15 years of age) and 2011 (18 years of age). The last visit (18 years of age) occurred between September 2011 and April 2012 and all members of the cohort were asked to appear at the Federal University of Pelotas for a clinical examination and the administration of questionnaires. Descriptions of the methods employed during past visits to the members of the cohort are found in previous studies [12], [13].

### Oral health subsamples of 1993 Pelotas birth cohort

The oral health follow up evaluations were performed with subsamples at six, 12 and 18 years. The subsample at six years was obtained from the follow up of the cohort performed when the children were one year of age, in which all underweight children were included. The oral health subsample at six years involved 359 members of the cohort, including 28.7% of underweight children. This information was weighted to represent the true proportion of live births of underweight children. Another home follow up was performed at 12 years, when 339 members of those visited at six years were located. The description of the oral health follow-up methods at six and 12 years is found in a previous publication [14].

The third follow up occurred at 18 years in a clinical setting during morning and afternoon shifts from Monday to Saturday from September 2011 to March 2012. The oral health subsample at 18 years was formed by adolescents who appeared for evaluations during the four predefined appointments (two in the morning and two in the afternoon) throughout the week, which were scheduled in a randomised fashion. Besides the adolescents who appeared for the predefined appointments, attempts were made to examine all 359 member members of the cohort that had been evaluated in the previous oral health subsamples. The oral health subsample of the final follow up evaluation consisted of 1014 adolescents, 307 of whom had been part of the subsample at six years of age and 301 of whom had been part of the subsample at 12 years of age.

### Oral health exams at 18 years of age

At the 2012 follow up, the oral health exam was performed by a single examiner who had undergone training and calibration exercises using the diagnostic criteria of the World Health Organization [15]. Two calibration exercises were conducted (August and December 2011) with the participation of the researcher of the study and three dentists. The aim of the two exercises was to maintain the reproducibility of the exams, as the fieldwork occurred over approximately eight months. Weighted Kappa coefficients were calculated. The lowest values during the two calibration exercises were 0.78 and 0.83 for intra-examiner and intra-examiner agreement, respectively.

### Outcome – Dental Caries

**Figure 1:**
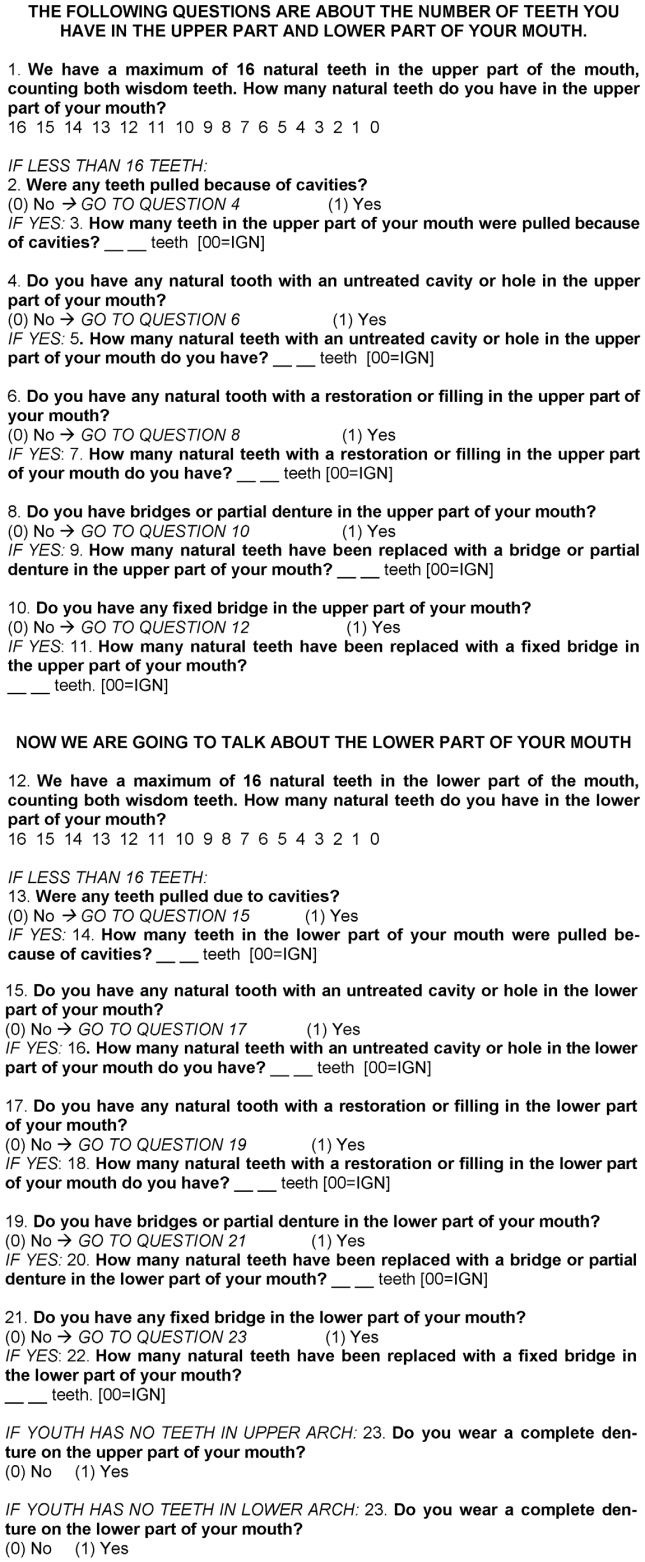
Self-reported survey of dental caries. Pelotas, Rio Grande do Sul, Brazil, 2012.

1. **Clinical dental caries.** A World Health Organization (WHO) periodontal probe and mouth mirror were used for the clinical detection of dental caries based on the WHO criteria [15]. The distal, vestibular, mesial, and lingual/palatine surfaces of the anterior teeth were examined. These same surfaces plus the occlusal surface were examined on the posterior teeth. The overall decayed, missing, and filled teeth (DMFT) index and each of its components were calculated for each participant. The prevalence of dental caries was determined based on the proportion of adolescents with DMFT ≥1.
2. **Self-reported dental caries.** For the self-reported data on dental caries, a 24-item questionnaire (Figure 1) was administered by trained interviewers to 4041 adolescents of the overall cohort. This questionnaire was first tested on individuals with a similar age to those in the cohort. The aim of obtaining self-reported information was to identify the individuals' knowledge regarding the number of decayed, missing and filled teeth. Based on the findings, the DMFT index was calculated for each participant. As with the clinical exam, the prevalence of dental caries was determined based on the proportion of adolescents with DMFT ≥1.

### Exploratory variables

A questionnaire addressing socioeconomic and demographic variables, visits to the dentist, tooth brushing habits, and self-rated oral health was administered to all adolescents in the cohort by the same interviewers who administered the questionnaire on self-reported dental caries. The demographic and socioeconomic variables analysed were gender, adolescent's schooling in number of completed years of study (categorised as ≤4, 5 to 8, 9 to 11, and ≥12), and household income in the previous month (sum of the monthly income of all members of the household in Brazilian currency [Real – R$], converted to a figure based on the minimum Brazilian wage and categorised in tertiles).

Variables related to oral health (visits to the dentist, daily tooth brushing frequency, and self-rated oral health) were obtained from the following questions: 1) Have you visited the dentist since <month> of last year (yes or no); 2) How many times a day do you brush your teeth? (categorised as <2 times a day and ≥2 times a day); and 3) How would you rate the health of your teeth today? (very good, good, fair, poor, and very poor [subsequently categorised as very good/good, fair, and poor/very poor]).

### Statistical analysis

The data were analysed with the aid of the Stata 12.0 program. The chi-square test was used to compare the participants in the subsample at 18 years of age with all members of the original cohort. Descriptive analysis was then performed of the DMFT index as well as the individual decayed, missing, and filled components, with the calculation of mean values and 95% confidence intervals (CI). The following were also calculated: intraclass correlation coefficient (ICC) for the DMFT index as well as the individual decayed, missing, and filled components; prevalence of clinically determined dental caries (gold standard); prevalence of self-reported dental caries; estimates of sensitivity, specificity, and positive and negative predictive values; absolute bias (prevalence of self-reported caries minus gold standard prevalence); relative bias (percentage of underestimation of true prevalence  =  absolute bias/gold standard prevalence × 100); and inflation factor (gold standard prevalence/self-reported prevalence) [16]. Estimates of sensitivity, specificity, and positive and negative predictive values were stratified by gender and schooling (12 or more years of study *versus* 4 years or less). Prevalence ratios for clinically determined and self-reported dental caries were calculated for risk factors using Poisson regression models with robust variance [17].

### Ethical aspects

This study received approval from the Human Research Ethics Committee of the Federal University of Pelotas (Brazil) under process number 67/11. All participants received clarifications regarding the objectives and procedures and agreed to participate by signing a statement of informed consent.

## Results

A total of 1014 members of the cohort were evaluated with regard to oral health at 18 years of age, 526 (51.9%) of whom were male and 386 (41.7%) had mothers with five to eight years of schooling. Regarding household income, the first, second, and third tertiles corresponded to less than two times the Brazilian minimum wage, between two and four times the minimum wage, and more than four times the minimum wage, respectively. Table 1 displays the characteristics of the original cohort (n = 5248) and oral health subsample (n = 1014). Statistically significant differences were found regarding gender, mother's schooling, and household income, with an increase in the proportion of male subjects, mean mother's schooling, and household income in the subsample.

**Table 1:** Characteristics of original 1993 birth cohort and oral health subsample of cohort members at 18 years age, Pelotas, Rio Grande do Sul, Brazil, 2012.

	Original cohort		Subsample at 18 years of ages		
	N	% (95%CI)	N	% (95%CI)	p-value *
**Gender**					
Male	2603	49.6 (48.3; 50.9)	526	52.2 (49.8; 55.3)	<0.001
Female	2645	50.4 (49.1; 51.8)	487	47.8 (44.6; 50.9)	
**Mother's schooling (years)**					
≤4	1468	28.0 (26.8; 29.2)	228	24.6 (21.8; 27.4)	
5–8	2424	46.24 (44.9; 47.6)	386	41.8 (38.5; 44.9)	<0.001
9–11	923	17.60 (16.6; 18.6)	261	28.4 (25.5; 31.4)	
≥12	427	8.1 (7.4; 8.9)	48	5.3 (3.8; 6.8)	
**Household income (based on minimum wage)**					
1^st^ tertile	2226	43.3 (41.9; 44.7)	339	32.7(29.9; 35.7)	
2^nd^ tertile	1445	28.1 (26.9; 29.4)	335	33.2(30.2; 36.2)	<0.001
3^rd^ tertile	1466	28.5 (27.3; 29.8)	336	34.0(31.0; 37.0)	

*chi-square test.

Both the clinical and self-reported DMFT indices had a median value of 1 decayed, missing or filled tooth and an interquartile interval (Q3–Q1) of 3 DMFT. Regarding the mean values, the clinical DMFT was 2.06, and the self-reported DMFT was 1.75. All mean values of the DMFT components demonstrated the underestimation of self-reported information in comparison to the clinical evaluation. In the analysis of the ICC, which allowed measuring the concordance or reliability of the tools used to compare the clinical and self-reported DMFT and its components, the lowest degree of reliability was found for the decayed component (ICC = 0.43) and the highest was found for the missing component (ICC = 0.61) (Table 2).

**Table 2:** Description and correlation of clinically determined and self-reported Decayed, Missing and Filled Teeth (DMFT) index of members of 1993 birth cohort at 18 years of age, Pelotas, Rio Grande do Sul, Brazil, 2012.

	Clinical Mean (95% CI)	Self-reported ean (95% CI)	Intraclass correlation coefficient	p-value
**DMFT**	2.06 (1.92; 2.21)	1.75 (1.68; 1.82)	0.50	<0.001
**Decayed component**	0.79 (0.71; 0.88)	0.63 (0.59; 0.67)	0.43	<0.001
**Missing component**	0.33 (0.29; 0.38)	0.28 (0.25; 0.30)	0.61	<0.001
**Filled component**	0.94 (0.84; 1.04)	0.84 (0.79; 0.89)	0.58	<0.001

Table 3 displays the prevalence rates of dental caries and estimates of sensitivity, specificity, positive and negative predictive values, absolute bias, relative bias, and inflation factor. Self-reports underestimated the prevalence of dental caries by 9.3% in comparison to the clinical evaluation. The results indicate high sensitivity (81.8%) and specificity (78.1%). The positive predictive value of 88% indicates that, among those identified with dental caries, the majority actually had the condition. The negative predictive value indicates that, among those identified as not having dental caries, only 68.6% were confirmed as actually not having the condition. The inflation factor for self-reported dental caries was 1.10.

**Table 3:** Prevalence of clinically determined and self-reported dental caries (DMFT ≥1) with estimates of sensitivity, specificity, positive predictive value, negative predictive value, absolute bias, relative bias and inflation factor for members of 1993 birth cohort at 18 years of age, Pelotas, Rio Grande do Sul, Brazil, 2012.

Prevalence of dental caries		
Clinical % (95% CI)	Self-reported % (95% CI)	p-value^1^
66.5(63.6; 69.3)	60.3(58.8; 61.8)	0,001

Dental caries determined by clinical (gold standard) and self-reported methods						
Sensitivity % (95% CI)	Specificity % (95% CI)	PPV^2^ % (95% CI)	NPV^3^ % (95% CI)	Absolute bias^4^	Relative bias^5^	Inflation factor^6^
81.8(78.7; 84.7)	78.1(73.3; 82.4)	88(85.2; 90.5)	68.6(63.7; 73.2)	−6.2	9.3	1.1

1 chi-squared test.

2 positive predictive value.

3 negative predictive value.

4 absolute bias  =  tested prevalence – gold standard prevalence.

5 relative bias  =  underestimated true prevalence  =  absolute bias/gold standard prevalence X 100.

6 inflation factor  =  gold standard prevalence/tested prevalence.

Table 4 displays the estimates of sensitivity, specificity, and positive and negative predictive values stratified by gender, greater (12 or more years of study) or less schooling (4 years or less), lower (1^st^ tertile) or higher (3^rd^ tertile) household income, and visit to the dentist in the previous year among the adolescents. Sensitivity was higher for female subjects (87.3%), adolescents with a greater level of schooling (82.6%), those with a lower household income (84.5%) and those who visited the dentist in the previous year (81.9%). Specificity was higher for male subjects (84.1%), adolescents with a greater level of schooling (92.6%), those with a higher household income (83.5%) and those who did not visit the dentist in the previous year (78.2%). The confirmation of the diagnosis of dental caries (measured by the positive predictive value) was also higher among male subjects (89.5%), adolescents with a greater level of schooling (90.5%), those with a higher household income (89.3%) and those who visited the dentist in the previous year (89.8%). The confirmation of the absence of the condition (measured by the negative predictive value) was greater among female subjects (71.6%), adolescents with a greater level of schooling (86.2%), those with a lower household income (69.7%) and those who did not visit the dentist in the previous year (72.5%).

**Table 4:** Estimates of sensitivity, specificity, positive predictive value and negative predictive value for clinically determined and self-reported dental caries (DMFT ≥1) according to gender, adolescent's schooling, household income and visit to dentist in previous year in 1993 birth cohort at 18 years of age, Pelotas, Rio Grande do Sul, Brazil, 2012.

	Sensitivity % (95% CI)	Specificity % (95% CI)	PPV^1^ % (95% CI)	NPV^2^ % (95% CI)	Accuracy
**Female**	87.3 (83.2; 90.7)	70.7(62.7; 77.8)	86.8 (82.7; 90.2)	71.6 (63.6; 78.7)	82.1
**Male**	76.4 (71.5; 80.9)	84.1 (78.1; 89.0)	89.5 (85.4; 92.8)	66.8 (60.4; 72.8)	78.2
**Lesser schooling of adolescent (≤4 years of study)**	75.0 (56.8; 88.5)	65.0 (40.8; 84.6)	77.4 (58.9; 90.4)	61.9 (38.4; 81.9)	71.1
**Greater schooling of adolescent (≥12 years of study)**	82.6 (61.2; 95.0)	92.6 (75.7; 99.1)	90.5 (69.6; 98.8)	86.2 (68.2; 96.1)	88.0
**Lower household income (1^st^ tertile)**	84.5 (79.8; 88.5)	75.4 (67.2; 82.4)	87.9 (83.4; 91.5)	69.7 (61.5; 77.0)	83.9
**Higher household income (3^rd^ tertile)**	78.0 (71.3; 83.8)	83.5 (74.9; 90.1)	89.3 (83.4; 93.6)	68.3 (59.4; 76.3)	80.0
**Visited dentist in previous year**	81.9 (77.6; 85.6)	78.0 (70.7; 84.2)	89.8 (86.1; 92.8)	64.6 (57.4; 71.3)	80.7
**Did not visit dentist in previous year**	80.8 (75.9; 86.0)	78.2 (71.4; 84.0)	85.9 (81.3; 89.8)	72.5 (65.7; 78.7)	80.4

1 positive predictive value.

2 negative predictive value.

Table 5 displays the prevalence ratios according to the independent variables evaluated for the binary DMFT (0/≥1) based on the clinical and self-reported assessments. Higher prevalence ratios were found for the majority of variables when considering clinically detected dental caries. Adolescent's schooling and household income were inversely associated with clinically detected dental caries. Self-rated oral health was positively associated with both the clinical and self-reported measures of dental caries. Clinically determined dental caries was positively associated with visits to the dentist in the previous year. The self-reported measure of dental caries was inversely associated with daily tooth brushing frequency.

**Table 5:** Prevalence ratios (95% CI) for exploratory variables of clinically determined and self-reported dental caries (DMFT ≥1) in 1993 cohort at 18 years of age, Pelotas, Rio Grande do Sul, Brazil, 2012.

	Clinical DMFT	Self-reported DMFT
**Gender**	**p = 0.003**	**p<0.001**
Male	1.0	1.0
Female	1.2 (1.1; 1.4)	1.25 (1.2; 1.4)
**Adolescent's schooling (years of study)**	**p<0.001**	**p<0.001**
≤4	2.1 (1.2; 3.6)	1.0 (0.9; 1.3)
5–8	2.5 (1.6; 4.0)	1.3 (1.1; 1.6)
9–11	1.8 (1.2; 3.6)	1.3 (1.0; 1.7)
≥12	1.0	1.0
**Household income (based on minimum wage)**	**p = 0.05**	**p = 0.27**
1^st^ tertile	1.2 (1.0; 1.5)	1.2(1.0; 1.4)
2^nd^ tertile	1.1 (0.9; 1.3)	1.1 (0.9; 1.3)
3^rd^ tertile	1.0	1.0
**Visit to dentist in previous year**	**p = 0.001**	**p = 0.098**
No	1.0	1.0
Yes	1.3 (1.2; 1.5)	1.1 (1.0; 1.3)
**Daily tooth brushing frequency**	**p = 0.81**	**p = 0.007**
<2 times	1.1 (0.7; 1.6)	1.3 (1.1; 1.6)
≥2 times	1.0	1.0
**Self-rated oral health**	**p<0.001**	**p<0.001**
Very good/good	1.0	1.0
Fair	1.4 (1.2; 1.6)	1.7 (1.4; 1.9)
Poor/very poor	2.1 (1.8; 2.6)	(2.6; 3.8)

## Discussion

The findings of the present study indicate adequate validity in self-reported data regarding dental caries in adolescents in relation to the clinical measure using the DMFT index. Self-reported measures exhibited high sensitivity and specificity.

To adjust the prevalence of self-reported dental caries (which was underestimated by 9.3% in comparison to the clinical evaluation in the present study), the literature proposes the use of correction factors, such as the inflation factor [16], for health surveys that assess self-reported information regarding periodontal disease [18].

Moderate agreement was found between self-reported and clinically determined tooth decay in the present study. Studies on the validation of self-reported oral health information have indicated low agreement regarding the comparison of the decayed component [2]. This may be related to the fact that laypersons are unable to recognise dental caries [2], [6] or only perceive the presence of the condition when it affects their social relations or when they experience pain [19].

Stronger agreement is described regarding reports of the number of missing and filled teeth [20]. The literature states that the number of self-reported missing teeth in adults and elderly individuals differs from the number determined clinically [10] because individuals have difficulties remembering treatment received years ago. Moreover, the loss of the first permanent molar may have occurred early in life, which further hinders the recollection of such an event. The stronger agreement in the present study regarding the number of missing teeth may be explained by the fact that adolescents are more likely to remember adverse oral conditions in their life. Moreover, missing teeth is a rare occurrence in this group. Indeed, the most recent national oral health surveys in Brazil [21], [22] report that adolescents between 15 and 19 years of age have an increasingly fewer number of decayed teeth, which is the main cause of tooth loss in this age group. This decrease in the prevalence of caries is directly related to an improved socioeconomic status, which allows the population access to fluoridated toothpaste, a fluoridated water supply and oral healthcare services [23].

The findings for the filled teeth component are in agreement with those reported in a previous study [2]. The greater agreement between the self-reported and clinical assessments regarding restored teeth is likely related to the aforementioned reduction in the prevalence of dental caries as well as the change in the oral healthcare model adopted in Brazil, which was previously directed more toward surgical and restorative procedures and currently involves a health vigilance model [24]. This aspect may have contributed to the fact that the adolescents analysed had an average of less than one tooth with carious tissue.

The similar prevalence rates between clinically determined and self-reported dental caries measured by the DMFT index in the present study were likely due to the fact that the clinical exam for dental caries based on the WHO criteria for epidemiological surveys identifies more advanced stages of tooth decay. Another aspect that diminished the possibility of error in self-reported information on DMFT was the fact that all participants were informed regarding the maximum number of teeth in the upper and lower arches, which differs from methods reported in previous studies [2], [5], [6].

The high sensitivity and specificity in the present investigation differ from findings reported in a study carried out in the United States involving individuals in different age groups (19 to 78 years), but the prevalence rate of dental caries was similar (63.8%) [2]. It should be stressed that the study cited only compared the decayed component, whereas the present investigation involved the entire DMFT index. Nonetheless, the difference between studies is likely due to the age groups analysed, which may have exerted an influence on the quality of the self-reported information, as discussed previously. Sensitivity is dependent on the prevalence of a disease [20]. Moreover, socioeconomic issues and health-related behaviour exert an influence on the quality of self-reported information regarding the health of a population and consequently affect the degree of sensitivity. The positive predictive value is affected by these same factors. In the analysis of adolescent's schooling, higher sensitivity and positive predictive values were found among those with a greater level of schooling and those who visited the dentist in the previous year. Thus, different results can be found in different socioeconomic contexts, indicating that the findings of the present study should be analysed with caution, considering the socioeconomic profile of Brazil.

In the analysis of the unadjusted prevalence ratios for socio-demographic aspects and oral health habits, some variables were only associated with the clinical determination of caries and others were only associated with self-reported caries. This indicates that self-reported information should be used with caution in epidemiological studies with the aim of establishing significant associations between risk factors and dental caries. None of the studies consulted in the literature performed such comparisons, with the exception of studies addressing self-reported information on periodontal disease [18].

The advantage of using self-reported information for knowledge on the situation of dental caries in adolescents resides in the fact that the data collection questionnaire can be administered by any trained interviewer and does not require a dental professional. For oral health professionals, especially those linked to public health-care services, such information is of extreme importance to the planning and monitoring of oral health policies and actions.

The quality of the information obtained in the present study should be stressed, as the data came from a sample from a cohort study, with due methodological care taken to confer a high degree of reliability to the information obtained.

Based on the present findings, self-reported information on dental caries using the DMFT index requires further studies prior to its use in the analysis of risk factors, but is valid for population-based health surveys with the aim of planning and monitoring oral health actions directed at adolescents without the need to submit the population to clinical exams for the diagnosis of dental caries.
